# Methylenedioxymethamphetamine-assisted Psychotherapy For Posttraumatic Stress Disorder: A Review

**DOI:** 10.1192/j.eurpsy.2022.1730

**Published:** 2022-09-01

**Authors:** J. Sá Couto, B. Da Luz, J. Rodrigues, M. Pão Trigo, T. Ventura Gil

**Affiliations:** Centro Hospitalar Universitário do Algarve, Departamento De Psiquiatria E Saúde Mental, Unidade De Faro, Faro, Portugal

**Keywords:** Psychotherapy, posttraumatic stress disorder, METHYLENEDIOXYMETHAMPHETAMINE

## Abstract

**Introduction:**

Posttraumatic stress disorder (PTSD) is a psychiatric condition which can be developed following traumatic experience. Treatment guidelines have long considered psychotherapy as a first line treatment. Despite that, PTSD remains an illness with high rates of comorbidity. Therefore, exploring novel therapies is of utmost importance.

**Objectives:**

Clarifying methylenedioxymethamphetamine (MDMA)-assisted psychotherapy efficacy in symptom relief in people with PTSD. Explaining clinical MDMA mechanism of action. Assessing safety of MDMA clinical use.

**Methods:**

*PubMed* database search, with *“MDMA for PTSD”* keyword expression. 12 Articles published in the last ten years were selected among the 112 best matches. Reference lists of articles were reviewed to identify additional articles.

**Results:**

Mithoefer *et al.* (2010) carried out the first controlled clinical study with MDMA-assisted psychotherapy in people with PTSD. Twenty patients with treatment-resistant PTSD were selected. They were given either placebo or two or three sessions of MDMA. 83% of the experimental group no longer met the criteria for PTSD (mean remission lasted 45 months without further MDMA doses) compared with 25% of the placebo group. Further studies were also suggestive of improvements in treatment-resistant PTSD patients undergoing MDMA-assisted psychotherapy. MDMA may increase exposure therapy effectiveness, allowing patients to stay emotionally involved while revisiting past traumas without being overwhelmed by anxiety and fear.

**Conclusions:**

To date, MDMA-assisted psychotherapy studies demonstrated consistently positive results. However, they have been carried out with small groups of individuals. Therefore, larger trials should be conducted to assess MDMA’s efficacy and safety for it to become a licensed medicine.

**Disclosure:**

No significant relationships.

